# Fungal Infection Post Arthroscopic Meniscal Repair: A Rare Complication

**DOI:** 10.7759/cureus.33342

**Published:** 2023-01-04

**Authors:** Sai Krishna MLV, Nitin Chauhan, Ravi Mittal, Aritra Chattopadhyay

**Affiliations:** 1 Orthopaedics, All India Institute of Medical Sciences, New Delhi, IND

**Keywords:** cryptococcus, surgical site infection, meniscus, fungal infection, arthroscopy

## Abstract

Infections after arthroscopic procedures are rare. Infection due to fungal organisms is rarer and difficult to diagnose due to its insidious nature and chronic presentation but when neglected has devastating consequences. We present a 23-year-old immunocompetent adult post-arthroscopic meniscal repair with fungal surgical site infection. The patient underwent open debridement and was started on antifungals. His surgical wound healed and with physiotherapy he regained his full range of movement.

## Introduction

Despite all the precautions, surgical wounds are still associated with infections. Bacteria are the usual cause and, *Staphylococcus aureus* is the most common among them. In wounds contaminated with farm and environmental debris, fungal organisms have been the cause of infection [1].

Arthroscopic surgeries are associated with less morbidity because of small incisions and minimal soft tissue handling. But at the same time, they are not free of complications. Some of the complications that can occur with arthroscopic procedures are hemarthrosis, neurovascular damage, deep venous thrombosis, compartment syndrome, scuffing of the cartilage due to instruments, damage to other intraarticular structures, instrument breakage, fluid dissection with fistula formations and infections [2].

Of all the arthroscopic procedures, arthroscopic knee surgeries are the commonest. Post arthroscopic procedures infection is rare with incidence ranging from 0.3% to 1.70% and mostly due to bacteria. Infection due to fungal organisms is even rarer and difficult to diagnose due to their insidious nature and chronic presentation but when neglected has devastating consequences. Only a handful of such complications involving fungal organisms have been described post-arthroscopic procedures [3-8]. Here we present an immunocompetent young adult who underwent arthroscopic medial meniscal repair and presented with postoperative fungal infection.

## Case presentation

A young adult of 23 years presented to us with right knee pain after a twisting injury of the knee. His magnetic resonance imaging (MRI) showed a bucket handle tear of the medial meniscus. The patient had no significant past or family history. He was operated on for the same using arthroscopic inside-out repair with ethibond sutures. He was given a single dose of cefuroxime preoperatively, and the same antibiotic was continued for 24 hours after surgery. He was discharged on the second postoperative day. At the time of discharge, his wound was clean. After two weeks of follow-up, sutures were removed, and knee mobilization was started along with full weight bearing. After one week of suture removal, he developed fullness over the knee associated with febrile episodes. He presented to us five days after his complaints started with knee swelling, medial wound gaping, and discharge. His blood sample was sent for investigation, and a knee MRI was obtained.

His erythrocyte sedimentation rate (ESR) was 50 mm/hr, and total leucocyte counts (TLC) was 14,320/cu.microL., differential counts were neutrophils-89%, lymphocytes-5.8%, monocytes-4.7%, and absolute neutrophil count was 12,770/cu.micorL and C-reactive protein (CRP) was 16.5 mg/dl (Table 1).

**Table 1:** Blood investigations of the patient (pre-operative period).

Test	Results	Range
Total Leucocyte Counts (TLC)	14,320/cu.microL	4000 – 11000/cu.microL
Differential counts	Neutrophils-89%	Neutrophils 40 – 80%
	Lymphocytes-5.8%	Lymphocytes 20 -40%
	Monocytes-4.7%	Monocytes 2-10%
Erythrocyte Sedimentation Rate (ESR)	50 mm/hr	0-15 mm/hr
C-Reactive Protein (CRP)	16.5 mg/dl	0-5 mg/dl

**Figure 1:**
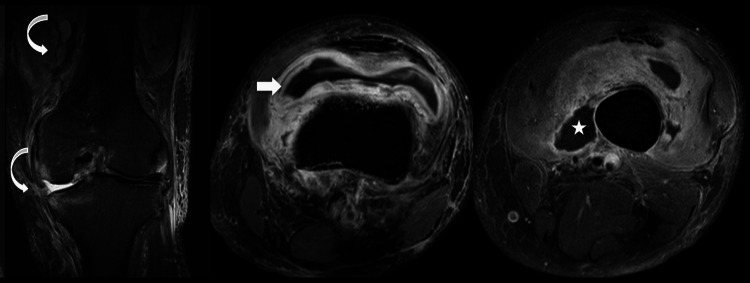
MRI of the knee The MRI was suggestive of synovial thickening (white arrow) and collections in lower thigh soft tissue (★). Also, there is extensive soft tissue edema around the knee joint and lower thigh region (curved arrow).

An MRI of the knee was done which was suggestive of synovial thickening and collections in lower thigh soft tissue. Also, there was extensive soft tissue edema around the knee joint and lower thigh region (Figure 1).

**Figure 2:**
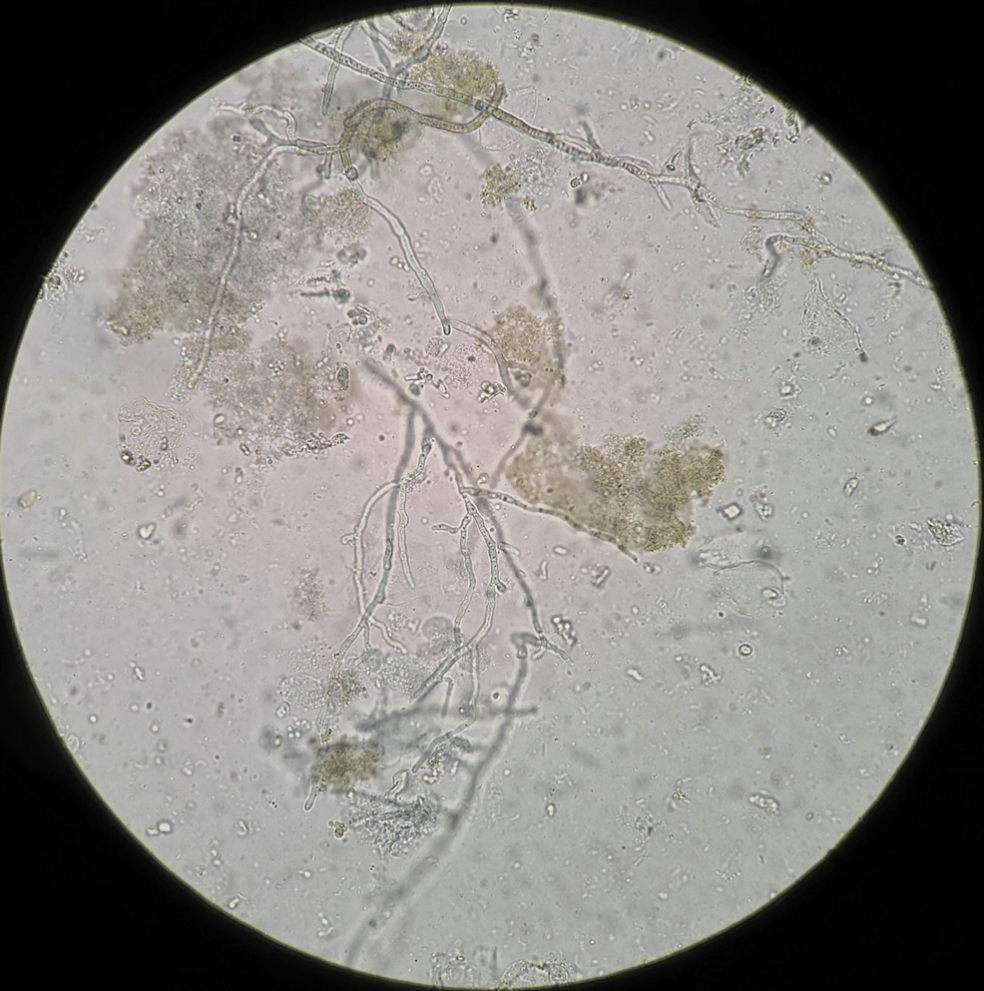
The KOH mount (40x) showed thin, hyaline, branched, septate hyphae. KOH: Potassium hydroxide

The patient was planned for an emergency arthrotomy. He was placed in a supine position and given regional anesthesia. Using a midline skin incision, a medial knee arthrotomy was done. There was unhealthy granulation tissue in the knee joint extending up to the anterior compartment of the thigh. Communication was seen with the medial surgical wound of the inside-out meniscal repair. Unhealthy granulation tissue and necrotic tissue were debrided. Thorough lavage was done with saline and the wound was closed over a drain. The drain was removed on the third postoperative day. His intraoperative debrided tissues were sent for cultures and histopathology. The direct potassium hydroxide (KOH) mount was positive for fungus wherein septate hyphae were seen (Figure 2). 

**Figure 3:**
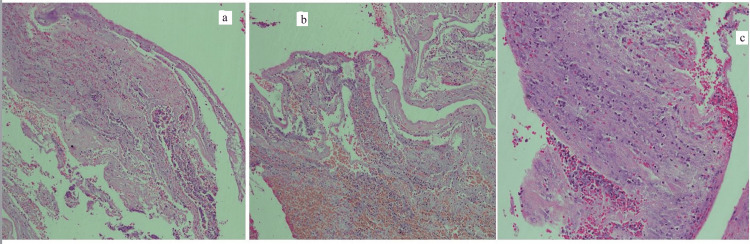
Histopathological examination images The histopathological examination was suggestive of fragments of synovial tissue with chronic inflammatory cell infiltrate (a), along with acute inflammatory exudate (b) and granulation tissue (c).

An infectious diseases consultation was sought, and he was started on voriconazole along with meropenem and teicoplanin as per their advice. Later when the bacterial cultures, tuberculosis polymerase chain reaction (TB PCR), and Genexpert test were reported as negative, meropenem and teicoplanin were discontinued. He was continued on voriconazole, and his blood drug levels were assessed regularly. His Galctomannan antigen test was negative (0.272). The histopathological examination was showing fragments of synovial tissue with chronic inflammatory cell infiltrate along with acute inflammatory exudate and granulation tissue (Figure 3).

Multiple specimens were sent for KOH mount, which were all positive for septate hyphae. But only one culture showed growth of *Cryptococcus gattii*. The blood *Cryptococcus* antigen test was negative. At five weeks post-operative period the TLC was 5130/cu.micorL, CRP was 3.7 mg/dl, and ESR- 38 mm/hr (Table 2). Voriconazole was continued for three months.

**Table 2:** Blood investigations of the patient (five weeks post-operative period).

Test	Results	Range
Total Leucocyte Counts (TLC)	5130/cu.microL	4000 – 11000/cu.microL
Erythrocyte Sedimentation Rate (ESR)	38 mm/hr	0-15 mm/hr
C-Reactive Protein (CRP)	3.7 mg/dl	0-5 mg/dl

The patient was started on isometric quadriceps exercises after surgery. The knee range of movement was started along with full weight bearing after the drains were removed. His suture removal was done at three weeks. At five weeks he attained 90 degrees of knee range of movement with full extension. His wound was healthy. At eight weeks postoperative period he attained 120 degrees of active knee flexion and is still doing regular physiotherapy. At the end of three months, he attained his full knee range of movement and the wound was healthy.

## Discussion

Approximately around 150 fungal organisms are known to be pathogenic to humans and animals among the 100,000 fungal species described. Fungal bone infections are uncommon but their incidence in causing skeletal infections is on the rise. Mycotic bone infections are seen both in immunocompromised and immunocompetent subjects. In immunocompromised patients and in subjects with risk factors like diabetes, steroid intake, and chemotherapy, where the defenses of the patients were defective they are prone to fungal infections. They can be caused either by hematogenous dissemination or direct extension from the nearby focus. The infection ranges from an indolent course to a disseminated fulminant course [3,4].

Post-operative mycotic skeletal surgical site infections are rare and such infections after arthroscopic surgeries are even rarer [4-8]. The patients present with local signs of inflammation and fever which are insidious. The inflammatory reaction or response due to fungal infection is less severe than that of pyogenic infection and the local inflammatory symptoms are relatively mild. There is no response to empirical antibiotics. And it might present at a later stage when substantial bone destruction has occurred. The treatment of such infections is similar to those of pyogenic surgical site infections. It involves debridement either arthroscopic or open. This is followed by antifungal drugs based on the KOH mounts, cultures, and biopsy reports. The cultures require a longer time and might yield negative results [3-8].

In our patient, post debridement the tissue from multiple sites was sent for gram stain and culture, acid-fast bacillus (AFB) stain, and fungal stains. All the specimens for the gram stains and the AFB stain were negative. The initial KOH mount revealed septate hyphae, and accordingly, he was started on voriconazole. All the KOH mounts were suggestive of septate hyphae, but only one was positive for culture at five weeks and was reported as *C. gattii*. The blood was negative for cryptococcal antigen, and the patient was responding to the oral antifungal treatment with no systemic symptoms. So as per the infectious disease consult, he was continued on voriconazole for three months.

Cryptococcus is encapsulated yeast that has two species, *C. neoformans* and *C. gattii*. *C.gattii* can cause infection in immunocompetent individuals. The route of infection is by inhalation of the spores which is usually asymptomatic and results in disseminated disease in severe cell-mediated immunodeficiency. Skeletal cryptococcal infections can occur as a part of disseminated disease or in isolation. Skeletal cryptococcosis in immunocompetent individuals is rare and many of them might have predisposing factors like tuberculosis, steroid intake, malignancy, chemotherapy, and diabetes mellitus. The diagnosis includes identification of the organism in the lesion and at the same time excluding systemic dissemination so that appropriate therapy could be started [9]. Our patient had no prior significant medical history and no comorbidities. He responded to oral antifungals and there were no systemic symptoms.

## Conclusions

Being an extremely rare complication, orthopaedic surgeons should have a suspicion of potential fungal infection as a cause of postoperative skeletal infection. Mycotic surgical site infections can also present in minimally invasive surgical procedures like arthroscopic procedures and even in young active immunocompetent patients. In postoperative surgical site infections, the debrided material should always be sent for fungal cultures and biopsies along with routine bacterial cultures.
